# Salutogenic model of health to identify turning points and coping styles for eating practices in type 2 diabetes mellitus

**DOI:** 10.1186/s12939-020-01194-4

**Published:** 2020-06-01

**Authors:** C. M. M. Polhuis, L. Vaandrager, S. S. Soedamah-Muthu, M. A. Koelen

**Affiliations:** 1 Health and Society, Wageningen University, P.O. Box 8130, 6700 EW Wageningen, The Netherlands; 2 Center of Research on Psychological and Somatic disorders (CORPS), Department of Medical and Clinical Psychology, Tilburg University, Tilburg, The Netherlands; 3 Institute for Food, Nutrition and Health, University of Reading, Reading, UK

**Keywords:** Everyday life, Dietary habits, Healthy eating, Coping, Salutogenesis, T2DM, Turning points, Stress, Mental health, Well-being

## Abstract

**Background:**

It is important for people with Type 2 Diabetes Mellitus (T2DM) to eat healthily. However, implementing dietary advice in everyday life is difficult, because eating is not a distinguishable action, but a chain of activities, embedded in social practices and influenced by previous life experiences. This research aims to understand why and how eating practices are developed over the life-course by investigating influential life experiences – turning points – and coping strategies for eating practices of people with T2DM.

**Methods:**

The Salutogenic Model of Health guided the study’s objective, study design and analysis. Seventeen interviews were performed and analysed based on the principles of interpretative phenomenological analysis. Narrative inquiry and the creation of timelines and food boxes were used as tools to facilitate reflection on turning points and eating practices.

**Results:**

Turning points for unhealthier eating were experiences that strongly disturbed the participants’ emotional stability. These experiences included psychosocial trauma, physical health disorders, job loss, and smoking cessation. Turning points for healthier eating were experiences that significantly changed participants views on life and made participants reflective about the effects of current eating practices on future health and life goals. These turning points included confrontation with ill-health, becoming a parent, psychosocial therapy, and getting married. Notably, turning points for healthier eating seemed only to happen when life was relatively stress-free. All participants experienced turning points for healthier eating, yet, not all participants succeeded in improving their diets. Two coping styles were distinguished: active and passive coping. Active coping individuals were able to act in line with their personal intentions, whereas passive coping individuals could not. Differences between active and passive coping styles seemed to be explained by differences in available resources important for adapting and maintaining a healthy diet.

**Conclusion:**

Disadvantaged childhood and later life adversities together with the inability to manage the mental stress explained the development unhealthier eating practices. All participants experienced turning points for healthier eating that caused eating to become a priority in their life. Yet, the fact that not all were able to eat as they intended, advocates for nutritional guidance for people with T2DM, with a greater emphasis on reflexivity, psycho-social well-being and social support.

## Introduction

Poor dietary habits are responsible for more deaths than any other risk factor globally, including smoking [1]. National nutrition surveys show that the majority of people do not follow dietary recommendations [2–5], which is one of the reasons why the prevalence of Type 2 Diabetes Mellitus (T2DM) has reached epidemic proportions globally. Current projects suggest that T2DM prevalence will reach 700 million people by 2045 [6]. In the Netherlands, 1,186,400 adults had diabetes in 2018, and it is expected that this number will rise to 1,320,000 adults in 2045 [7, 8]. Incidence is particularly high in people with low socioeconomic position [9, 10]. Once diagnosed, there is a strong emphasis on adopting a healthy diet [11]. Healthy eating can drastically improve glycaemic control, and in some cases, reverse the disease [12–16]. However, individuals with T2DM have indicated that committing to a healthier diet in everyday life is the most complex aspect of self-management [17–20].

Reasons for the complexity experienced may be in regard to gaps in knowledge of biomedical understandings of healthy eating and daily practices. Indeed, research has suggested that lay individuals and health professionals often speak different languages when discussing health and diet [21–24]. Healthcare professionals commonly work within a biomedical paradigm in which taking care of one’s diet is seen as an individual’s responsibility. One should eat according to national dietary guidelines: no alcohol, lower intake of foods containing saturated fats, sugars and salt, and higher intake of foods containing unsaturated fat and fibre (fruits, vegetables, legumes) [11]. However, in everyday life, healthy eating goes beyond the understanding of a good balance of macro- and micro-nutrients; it is also about structure and regularity in eating (e.g. eating a fixed number of meals at fixed times, or weekly routines), how foods are produced (e.g. home-made or organic), and psychosocial well-being (enjoying foods together) [21–24]. Eating is highly contextual, and personal interpretations of healthy eating are complex and diverse, as they reflect personal-, social-, and cultural experiences, as well as local (food) environments [23]. Besides the social context, eating practices are also embedded in a temporal context. Past experiences direct how people make food choices in the future [23, 25, 26]. Evidence shows that meanings of and attitudes towards healthy eating can change over time and are specific to life stages [23]. For example, being married and having a young child has been associated positively with fruit and vegetable consumption [27].

In addition, eating practices can also drastically and suddenly change after experiencing a turning point [25, 28]. Turning points are generally defined as powerful emotional or existential experiences that lead to relative drastic changes (in eating practices) that involve self-redefinition and changes in ego-identities, from which people do not turn back [25, 28–30]. The transition to motherhood has been indicated as a turning point for instance [31, 32]. Some recent research has observed that existential experiences influenced self-management behaviours among people with T2DM [29, 30, 33, 34]. For example, distressing evidence about one’s health led to small behavioural change action steps [29, 30, 33] as well as experiencing an ‘a-ha’ moment – a realisation that a particular self-management strategy actually worked – at critical points [34]. Nevertheless, the understanding of turning points for eating behaviour is still limited [25].

**Fig. 1:**
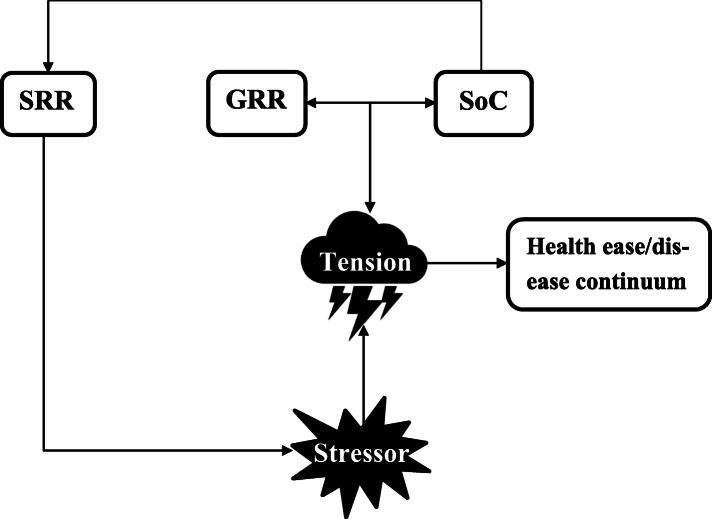
Simplified visual representation of the Salutogenic Model of Health (adapted from [37]; p. 184–185). How an individual copes with the tension created by a stressor is the result of the strength of SoC (i.e. capability to identify and mobilise S/GRRs) and the availability of GRRs. Via the SoC, GRRs determine the extent to which SRRs are available. A SRR is a resource that is activated specifically to cope with a specific stressor. The strength of SoC and availability of GRRs and SRRs leads to successful or to unsuccessful tension management, which eventually determines someone’s position on the ease-disease continuum

As indicated, the everyday life understanding of healthy eating overlaps but is not synonymous, with the biomedical understanding [35]. Furthermore, the fields of public health and medicine have been gradually shifting from a sole focus on the individual-level models to a greater focus on socioecological models [36]. A theoretical model that is both closer to the everyday life understanding of healthy eating and has incorporated a socioecological approach is the Salutogenic Model of Health (Fig. 1). The Salutogenic Model is centred around the idea that health results of continuous everyday life interactions between the individual and inevitable social-, economic-, cultural-, physical-, mental- and biochemical stressors [38]. The availability of resources that promote health and the capabilities in identifying and using these resources for overcoming tensions determines if health deteriorates, is sustained or is gained [37]. The individual capability to identify and mobilise resources is called the Sense of Coherence (SoC) and resources that promote health and facilitate coping with stressors are called Generalized Resistance Resources (GRRs). The SoC can be quantitatively measured and a stronger SoC is associated with better (mental- and physical-) health [39, 40] and healthier eating [41–43]. Regarding T2DM, a higher SoC is associated with better metabolic control [44, 45]. Originally, it was thought that SoC was a stable entity that developed mostly during the first decades of life and stabilised thereafter [37], however, more recent evidence shows that influential life experiences [46] – turning points – and even interventions [47–51] can alter SoC later in life. GRRs can be genetic-, material-, constitutional- and/or psychosocial resources. GRRs determine the extent to which Specific Resistance Resources (SRRs) are available [52]. SRRs are useful in *specific* situations of tension [37]. The idea is that if SoC and GRRs are well-developed, it facilitates identification of SRRs and development of coping strategies for specific challenges, in this case, for healthier eating. Cues from the situational context may cause that individuals are suddenly able to recognize and mobilize resources important for healthful eating. This idea is widely accepted in other conceptual health psychology and coping models as well (e.g. social learning theory [53], coping theories [54], health belief model [55], teachable moment framework [56]).

In this research, life-course interviews are used to explore how and why the eating practices of adults with T2DM (and low socioeconomic position in particular) are developed over time by investigating the everyday life experiences that led to key turning points for eating practices and coping strategies for healthier eating. By adopting a constructivist view, a turning point was considered as turning point when the *participant* considered and mentioned it to be a turning point. The results extended the knowledge on turning points for eating practices, and on learning experiences significant for developing the SoC. This is relevant for realising more effective personalised nutritional therapy for people with T2DM.

## Methods

### Participants

General Practitioners (GPs), practice nurses, and dieticians recruited people with T2DM in the province of Gelderland in the Netherlands between April and September 2018. Individuals that met the following criteria could participate: 1) low socioeconomic position; 2) native Dutch; 3) officially diagnosed with T2DM for at least 6 months; and 4) aged > 18 years. Adults with T2DM and low socioeconomic position were of interest, because T2DM is most prevalent among people with low socioeconomic position in the Netherlands [57], but at the same time, people with low socioeconomic position are underrepresented in research [58]. It was decided to focus on native Dutch people, as they generally share the same cultural- and historical backgrounds, are accustomed to similar products and food traditions, and have the same accessibility to food and healthcare. People had to be diagnosed for at least 6 months to ensure having sufficient experiences with dealing with T2DM in everyday life. People with cognitive disorders (e.g. dementia, or intellectual impairment) could not participate. Socioeconomic position was assessed by a researcher during the introductory meeting, as the recruiters were not comfortable asking about this. Twenty individuals were approached, 17 provided informed consent and participated in this study. Reasons for non-participation were not showing up in the introductory meeting (*n* = 1) and issues with scheduling the interviews (*n* = 2).^1^ Data saturation was reached after 13 interviews. Four additional interviews were conducted because these interviews were already scheduled at that point. The study was approved by the Social Ethical Committee of the Social Sciences Department of Wageningen University.

### Methods and materials

#### Timeline and food box

The study is based on principles of Interpretative Phenomenological Analysis (IPA). IPA was chosen because the ontological and epistemological positioning is similar to the Salutogenic Model of Health. Both view reality and people as inseparable; individuals are not viewers of the world, but are embedded in it [59]. Both also consider everyday life experiences as valuable sources for expressing the complexity and depth of human existence. IPA is a qualitative framework that is specifically suitable for analysing the meaning of everyday life experiences [60, 61]. In IPA, time and space are conceptualised that time is experienced as *temporal* and space is *situated* [59]. Temporality refers to how our past experiences direct how we are in the present. Situatedness does not refer to the geographical features of someone’s world, but to the experiences (situations) that are brought into the foreground by someone because they are the most meaningful to that person [59].

Inspired by the study of Swan et al. [62], narrative inquiry, systematic listening to people’s life stories, was used to facilitate a deep conversation on life-course experiences that influenced eating practices and to gain inside perspective of the personal life-worlds of participants. For this, the participants were asked in advance of the interview to create a timeline (from birth to present), in which they had to indicate turning points that changed their diet drastically, and construct a food box by collecting meaningful items (foods, but also non-edible objects, such as photos or utensils, were allowed) in a box that represented current eating practices. Timeline and food box are established research tools for sharing and reflecting upon experiences [62, 63]. In contrast to more structured interview methods, timelines and food boxes allow participants to tell their story in their own way, and steer the conversation to topics that are most important to them (*situatedness*), which facilitates the process of opening up on loaded/personal subjects, such as eating and weight. While the timeline was used to construct a *temporal* narrative on how eating practices had evolved, the food box was used to start the conversation on current eating practices. Hence, the timeline and food box were used as tools to facilitate reflection.

#### Protocol and measures

The researcher (CMMP) organised an introductory meeting with each participant individually to explain the research interests and procedure and to hand out the materials for the timeline and the food box. After signing the informed consent, questions related to demographics (age, living situation), socioeconomic position (income, educational level, occupation status), T2DM (T2DM duration, latest **glycated haemoglobin (HbA1c)** level and/or fasted glucose level), **and** self-care management (physical activity, self-monitoring blood glucose, and smoking [64]) were asked. Individual SoC was also quantitatively assessed with the SoC-13 [65]. The SoC-13 is a validated questionnaire with a scale of 0 to 52. Some participants needed assistance with filling in the questionnaires due to difficulties with reading and/or writing (*n* = 4). The introductory meeting was also set to establish a relationship with the participant in advance of the interview.

One week after the introductory meeting, the in-depth interview took place. All interviews were conducted in Dutch. The interview began by asking the participant to explain his/her timeline from birth to present and how the turning points influenced eating practices. Secondly, the participant was asked to explain why (s)he had chosen the specific objects in the food box. The researchers probed with questions when they wanted the participant to describe events or coping strategies in more detail. The participant was stimulated to take the lead during the interview. Exemplar questions that were asked frequently are summarised in Table 1.

**Table 1:** Key interview questions and examples of follow-up questions

	Timeline	Food-box
**Key questions**	Could you talk me through your timeline? In what way did this specific moment change eating practices/behaviour/diet? What were the things you were eating during this specific period?	Could you explain why you have chosen these specific objects?
**Sample follow-up questions**	How was your childhood? Could you describe how things used to be at the dinner table when you were a child? What kind of foods did you eat as a child? How did you learn how to cook? When did you leave parental house? What kind of foods are liked by your partner? When did you become a parent? What is it like being a parent? How did you experienced breakfast/lunch/dinner when your children lived at home? What foods did/do your children like? How did T2DM diagnosis affect you? How has T2DM diagnosis influenced your eating behaviour?	When did you start eating this specific product? Do you eat/use this at specific occasions or with specific persons? Are you satisfied with your current eating practices? And why (not)? What things make it easier for you to eat healthily? Why? What things make it difficult for you to eat healthily? Why? How do you deal with these? What would be your ideal eating pattern? What would help you to reach this ideal eating pattern? If you compare your eating practices in the past to now, what has changed?

Interviews lasted on average 76 min, ranging from 55 to 104 min. Participants were rewarded a ten euro gift-voucher after the interview. Both the introductory meeting and the interview took place at convenient locations for the participant (participant’s home or the university). One week after the interview, the researcher called the participant and asked him or her to reflect on the interview. The participant’s GP would have been contacted if the researcher suspected emotional/psychosocial harm caused by the interview that required additional medical/psychosocial support (in none of the cases necessary). The interviews and follow-up calls were recorded on a hand-held digital voice recorder and were described ad verbatim by two research assistants.

### Analysis

The IPA approach concentrates on properly exploring, understanding and communicating the individual- and unique meaning of specific events within personal contexts [60]. The researcher has an active role in this. The researcher tries to get an ‘insider’s perspective’ of the participants world, but cannot do this directly nor completely. The access depends (and is complicated by) the researcher’s own interpretations. The researcher’s interpretation is *necessary* for the sense-making of the participant’s world [59, 66]. Even though IPA is more of a philosophical ‘stance’, from which qualitative analyse is approached rather than a distinct method, the following steps (based on [60, 66, 67]) were followed to unravel the evolvement of eating practices over the life-course.

Firstly, a transcript was read and re-read to engage with the data. The data consisted of the transcripts of the interviews. The timelines and food boxes were not used in the data analysis. The reason for this is that the conversations, not the timelines nor food boxes, provided insights in answers to our research question. Elaborating on the timeline and food box would distract from the main results. With the use of Atlas.ti (Version 8), the transcript was open-coded with notes about observations, comments and reflections. In IPA, notes are made about interesting or significant statements of participants [60]. There are no rules for what is commented upon (i.e. notes can be on a descriptive, conceptual, and linguistic level) [60]. In this study, coding was mostly on a descriptive (to let things speak for themselves) and interpretative level (to decode the underlying meaning of experiences). More specifically, notes included:

- Explanations of *why* this specific participant identified these specific events as turning points, and of *how* the social-, historical- and/or physical context influenced eating behaviour in turning points.
- Individual challenges to, coping strategies with, and resources for healthy eating
- The researcher’s impression of the participant’s character, and if (s)he seemed to be in charge of eating behaviour (i.e. eating in line with *intentions*, not necessarily with dietary guidelines) and life in general (i.e. is this person living his/her ideal life? Is (s)he happy with his/her life?).

Subsequently, the notes were clustered in preliminary themes. Each interview was independently analysed to completion *before* moving on with the next interview [67]. In the next stage, the preliminary themes were compared across the data set, identifiable themes were connected, and idiosyncratic differences were noted. In the final stage, the theory of salutogenesis was used to further elicit *why* turning points facilitated or challenged healthy eating by discussing how previous experiences, outlooks on life, and internal and external resources led to developing coping styles and strategies [65]. Data collection continued until data saturation was reached (i.e. no new information was observed in the data). All interviews were analysed independently by CMMP and (at least) one of the other authors, and were subsequently discussed until consensus was reached. The overarching themes were the result of various discussions between all authors. The themes are displayed with pertinent participant quotes and detailed interpretative commentary. The quotes were translated to English by a professional editor.

## Results

The first part of the results describes the participants characteristics. The second part describes the key findings regarding the turning points for unhealthy and healthy eating. Temporal and situatedness-related aspects of the turning points are discussed as well. The final part describes how and why people react differently to similar experiences. The distinctive coping styles for healthy eating are described as active and passive coping.

### Participants’ characteristics

Eight men and nine women participated in the study (Table 2). Each was given a pseudonym for the purposes of tabulating the results. The average age was 67.7 (SD = 7.1) years old, ranging from 49 to 77 years old. All, except one, had children. Most lived with a partner and/or children, four participants lived alone. The average T2DM duration was 12 (SD = 6.5) years and ranged between 0.5 to 23 years. Six participants were able to keep HbA1c and/or fasted glucose values below the recommend target values of 53 mmol/L and 6.9 mmol/L, respectively [68]. Four participants managed to be physically active for 30 min each day of the week. Six participants indicated that they regularly self-monitor blood glucose. One of the participants was a smoker; four participant have smoked in the past. The SoC was relatively high: more than half of the participants had a high SoC (*n* = 11). The average SoC score was 35 (SD = 9.1) and ranged from 21 to 49.

**Table 2:** Overview of the participants’ personal, socioeconomic position, T2DM, self-management and SoC characteristics

Personal characteristics				Socioeconomic position			T2DM			Self-management			SoC
*Pseudonym*	*Age (years)*	*Living situation*	*Children (n)*	*Education*^*1*^	*Income*^*2*^	*Occupation status*^*3*^	*Disease duration (years)*	*HbA1c*^*4*^*(mmol/L)*	*FGL*^*5*^*(mmol/L)*	*Physical activity*^*6*^*(days/week)*	*Self-monitoring blood glucose*^*7*^*(days/week)*	*Smoking*	*SoC*^*7*^*(0–52)*
Diane ♀	49	Children	2	Medium	Low	Low^c^	5	69	6.4	0	0	No^h^	21
Ria ♀	75	Partner	2	Medium	Medium^a^	Low^d, e^	10	–	7.0	7	0	No	30.5
Annie ♀	60	Alone	0	Medium	Low	Low^c^	11	52	6.8	3.5	0	No^h^	42
Mieke ♀	56	Partner + children	2	Medium	Medium	Medium	16	–	7.0	7	0	No	35.5
Saskia ♀	67	Alone	2	Low	Low	Low^d, e^	20	92	11.1	1.5	1–2	No	24
Karin ♀	65	Alone	2	Medium	Low	Low^d, e^	0.5	69	11.0	1.5	0	No	35
Jan ♂	73	Partner	1	Low	Medium	Low^d^	0.5	–	–	0.5	0	No	41
Carla ♀	71	Partner	1	Medium	Medium^b^	Low^d^	10	–	9.4^g^	1.5	1	Yes	49
Freek ♂	72	Partner + children	3	Medium	Low	Low^d^	19	62	–	3.5	0	No^5^	41
Marja ♀	69	Partner	1	Medium	–	Low^d^	10	43	8.5	3.5	0	No	21.5
Henk ♂	66	Partner	3	Medium	Low	Low^f^	10	60	–	7	0	No	26.5
Mark ♂	66	Partner + children	3	Medium	Low	Medium^d^	21	66	–	7	7	No	43
Dennis ♂	69	Partner + children	3	Medium	Medium	Low^d^	10	–	5.5	3.5	0–1	No	36
Claudia ♀	77	Alone	2	Low	Low	Low^d^	15	–	6.4	1	0–1	No	22
Tygo ♂	71	Partner	2	Low	Low	Low^d^	10	–	–	5	0	No^h^	44
Theo ♂	62	Partner + children	4	High	High	Low^f^	10–12	–	7.2	0	0	No	43
Robert ♂	64	Partner	2	Medium	Low	Low^f^	23	61	–	3.5	1	No	40

^1^Based on the highest completed education. **Low education**: Primary education; **Medium education**: Basis secondary education (Junior secondary pre-vocational education, junior secondary general education, secondary general education, pre-uni versity education, senior secondary vocational education (Known as VMBO, VBO, MAVO, HAVO, VWO, MBO in Dutch); **High education**: Higher professional education or academic higher education (university) (Known as HBO or WO in Dutch); **Low education:** Primary education

^2^ Based on self-reported current net monthly income. Categorisation of incomes is based on the average net income in the Netherlands (i.e. net income of 2120 euro/month (CBP 2019)). **Low income**: < 2120 euro/month; **Medium income**: 2000–2500 euro/month; **High income**: > 2500 euro/month

^3^ Based on current occupation status. When retired, the classification was based on the latest occupation. **Low occupation status**: Unemployed, medically declared unfit for work, or occupations that do not require secondary education; **Medium occupation status**: Occupations that require medium education; **High occupation status**: Occupations that require high education Occupations that require medium education; **Low occupation status:** Unemployed, medically declared unfit for work, or occupations that do not require secondary education

^4^ HbA1c are self-reported. Cut-off values are based on Diabetes Fonds [68]: **Low HbA1c:** < 53 mmol/L; **Slightly alleviated HbA1c:** 54–63 mmol/; **Alleviated HbA1c:** 64–85 mmol/L; **High HbA1c:** > 86 mmol/L

^5^**FGL =** Fasting glucose levels; self-reported. Cut-off values are based on Diabetes Fonds [68]: **Low FGL:** < 6.1 mmol/L; **Medium FGL:** 6.1–6.9 mmol/L; **High FGL:** > 6.9 mmol/L

^6^ Physically active for at least 30 min

^7^ SoC-13 total score = 52. **Low SoC:** < 17; **Medium SoC**: 18–35; **High SoC**: > 35. Cut-off values are based on 52/3 = 17

^a^ Husband’s pension ^b^ Combined pensions (husband’s and wife’s) ^c^ Unemployed ^d^ Currently retired ^e^ No professional career; housewife ^f^ Medically declared unfit for work ^g^ Measured in non-fasting state ^h^ Past smoker

**Table 3:**
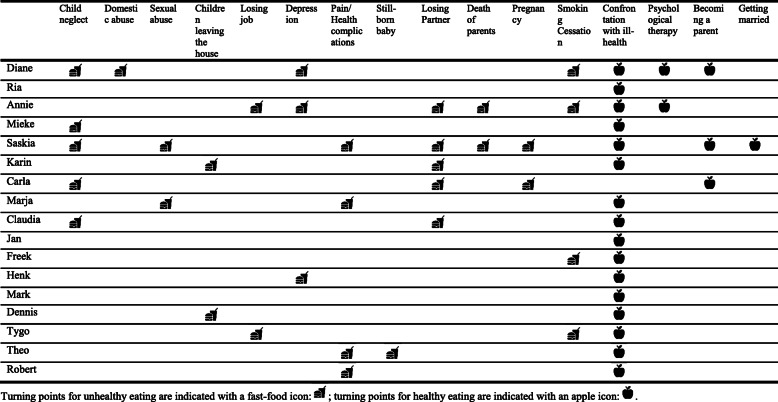
Overview of all identified turning points

Turning points for unhealthy eating are indicated with a fast-food icon: ; turning points for healthy eating are indicated with an apple icon:

### Turning points

A total of 15 different type of turning points were identified (Table 3). Turning points that led to unhealthy eating are identified with a fast-food icon () and turning points that led to healthy eating with an apple icon (). The participants that experienced turning points for healthy as well as unhealthy eating, encountered generally more turning points for unhealthy eating than for healthy eating (Table 3). Some participants only identified turning points for healthy eating (Ria, Mieke, Jan, and Mark). For them, unhealthy eating was a matter of a gradual worsening of eating practices caused by specific life phases (i.e. work, marriage, having children, etc.) and lack of resources (i.e. disadvantaged childhood, lack of nutritional knowledge), rather than sudden changes caused by isolated events.

#### Turning points for unhealthy eating

Turning points that induced unhealthy eating were experiences that disturbed the participants’ emotional stability through strong feelings of grief, loneliness, being out of control, and/or mental stress. The turning points ranged from traumatic experiences (childhood neglect; loss or sickness of loved ones; sexual abuse; domestic abuse), physical health problems (complicated pregnancy; pain; onset of chronic illnesses), mentally draining conditions (depression/burn-out), job loss, and smoking cessation. This stress was so overwhelming at the time that it required the full capacity of the participants’ consciousness and coping abilities to carry on with ‘normal’ everyday life (i.e. work, taking care of family). Diet and the impact of food choices on (long-term) health became simply secondary to these overwhelming events.

**Saskia:** It’s just that worries about my daughter [*who was receiving care from a psychiatric institute*] were a priority [*above healthy eating*]. And those worries just dominated my life- the anxiety and everything [...] Stress had a big influence on me at that time. […] If I am tense.. well, if I am tense and realise that, I really don’t have to check my glucose, then I know that I am on a level of 13, 14 and sometimes 15 [*blood glucose level*]. When I am more rested, I can think more clearly, and when I can accept my situation, then I know that just go back again to a level of 8.

Participants found it difficult to regain stability over their emotional states in the context of everyday life. This manifested itself, in some instances through the participant not paying attention to what was eaten, sometimes by the participant eating very little (e.g. Carla), but mostly by participants eating excessively (e.g. Tygo). In case of the latter, excessive eating was used to progress and regain command of their (negative) emotions (i.e. emotional eating).

**Carla:** [*Who talks about her partner leaving her and cheating her financially*] With that experience, I had such an emotional knock, I was like this [*pointing her index fingers together*]. I lost so much weight then. And I really couldn’t eat or drink. Nothing. [...] My boys went to my mother [...], because I didn’t cook. I didn’t feel like it at all.

**Tygo:** I am an emotional eater, if I feel bad, I eat everything I can get my hands on.

For example, losing a loved one, especially when it involved a spouse, caused loneliness, which led to less interest in cooking, and sadness, which led to emotional eating (e.g. Karin, Saskia).

**Karin:** I actually think that after my husband died, I started eating a lot due to grief. I would describe this as, responding through snacking. Then the weight piled on. Now that I think about it. Before that, it was different. When you have a partner, you don’t sneakily snack. At least, I didn’t.. but when you are alone [...] and you experience grief, you don’t know what you have to do to cope with it. He always came home at 3 o’clock [*in the afternoon; a typical time for tea/coffee in the Netherlands*] [...] Well, now, I do have a cookie or something with may tea, but I used to have a nice chat with him. Now I have to drink my tea or coffee alone, you know. It is actually that I started to eat to cope with the grief.

**Saskia**: That’s when I started injections, after he [*husband*] died. The anxiety, the stress, are cooped up inside me.

The onset of certain physical health problems caused stress, which led to unhealthy eating, because it confounded daily life through feelings of social isolation (i.e. in the instance that a participant was declared medically unfit for work), impediment of living an active healthy lifestyle, and chronic suffering from pain (e.g. Parkinson’s disease, hernia, fibromyalgia). Excessive eating was also used to cope with the stress caused by smoking cessation. Commonly, the participants experienced a lack of social support while dealing with the emotional aftermath of turning points for unhealthy eating.

#### Turning points for healthy eating

Turning points that induced healthy eating were experiences that significantly changed participants views on life and made participants reflective about the effects of current unhealthy eating practices on future health and goals. These turning points included confrontation with ill-health, becoming a parent, psychosocial therapy, and getting married. It is important to emphasise that these turning points generally happened in late-adulthood when most participants were in comparatively *‘calmer waters’* in terms of life circumstances (e.g. retirement; financial stability) and mental well-being (e.g. a current stress-free state-of-mind, being loved/supported by family/friends).

**Diane:** Yes. I have recouped after hitting ‘rock bottom’. I do notice that I manage my life more than before. That being healthy, and that quitting smoking also came up as an issue. I just succeeded with that in one go.

Secondly, it is important to note that turning points for healthy eating did not always have an infinite effect on eating practices. For example, Robert and Saskia managed to achieve significant weight loss (30–45 kg), but unfortunately regained much of it later due to other health complications (Parkinson’s complicated Robert’s strict lifestyle regimen; stomach operations and infections led to the need to remove Saskia’s gastric band).

A confrontation with ill-health was the most frequently identified turning point, which also caused the most drastic and long-lasting dietary improvements. In some cases, the reason was external, because it came from health professionals (e.g. T2DM diagnosis, warning about high blood glucose values), or from family/friends (e.g. pressure from Robert’s son to follow a lifestyle program; the advice of Tygo’s fitness trainer to consult a dietician). In other cases, the reason was internal, because participants themselves noticed alarming cues: Claudia decided to start cooking more frequently after just feeling unwell, Henk started dieting after being shocked at his high weight, Theo decided to limit his alcohol intake after realising it was getting out of control, and Mieke took better care of herself after being shocked by the death of her mother. Participants improved their diets either on their own, or with help of a dietician, by following a lifestyle program, or by undergoing bariatric surgery.

**Henk:** Not being entirely in control. Your body always craved food. And then it ended and now, I have it reasonably under control.

**Interviewer:** Yes, and how did you succeed?

**Henk:** Through shock. At 83 k, I realised that it was going wrong. And then other things came to light.

Secondly, becoming a parent was identified as turning point by Diane, Saskia and Carla. Although most participants had children and generally they indicated that this only changed eating practices *slightly*, Diane, Saskia and Carla stressed that parenting has improved their eating practices drastically. Unlike the other participants with children, Diane, Saskia and Carla experienced parental abandonment and child neglect in their own childhoods. Their parents were emotionally unavailable, and due to either financial- or health reasons, were unable to provide food and enjoy foods together with their children. Being parents themselves gave their life a new sense of purpose that strongly motivated them to do things better. This included providing healthy meals, and enjoying meals together with their kids, but also taking better care of their own diets and weight, because they wished to stay as healthy as possible for as long as possible.

**Diane:** That first year, before I had children [*I didn’t cook*]. That time I associate with really living for fun. I was drunk almost every day [*laughing*]. Yes, that was quite something.. yes, wild! [...] I also sometimes used to say that she [my daughter] saved my life.

**Interviewer:** How did you go about [*Eating more healthily*]?

**Diane:** Oh I just did. I knew clearly how it shouldn’t be, but I didn’t know exactly how it should be. And yes. Making the most of it […], but that is with everything. Also, with raising the children. I was not raised properly. That’s way I needed assistance with raising them.. that’s also how it is with cooking. However, I enjoyed having breakfast together in the morning [...] I thought that was always very important.

Thirdly, psychosocial group therapy was identified as a turning point by Diane and Annie. After suffering from depression, psychosocial therapy changed their outlook on life and equipped them with tools to take better care of their mental well-being. They emphasised that the most helpful aspect of their therapy was sharing their stories with others and feeling understood. Once their mental health was improved, there was more room to take better care of their diets as well.

**Annie:** I have experienced it as a very wonderful experience. Due to the fact that you are sitting with a group of people, around 10, 12 people, who actually know very well what you are going through, because you are almost all going through the same thing, and that makes it easier to live with. You don’t have to explain anything. [...] I still have contact with a few people from there. [...] You are, of course, receiving therapy at that time, and at some point, you start to see things differently. And then you have slowly elevated your perspective [...] this comes from how they support you in looking at things differently. You realise that the outlook is simply different. [...] And I have to say, I have benefitted a lot from it.

Finally, Saskia identified the prospect of getting married as a ‘small’ turning point. Her wedding motivated her to lose weight as quickly as possible. Looking thin was important for her, because she had felt always ashamed of her weight and appearance, as she had been overweight as a child.

**Saskia:** Before getting married [...] I had also lost a lot of weight. [...] I wanted to fit into my wedding dress [...], but that motivation maybe lasted a week after that. After getting married, it was gone again [...] That now seems simply unwise: [...] to make sure you were slim as possible just to fit into your wedding dress.

#### Personal meaning of turning points

Although parallels and commonalities are described among the different types of turning points, it should be stressed that consideration of the personal connotations to the individual of the turning point, together with contextual- and the temporal aspects is needed to fully understand *why* a specific experience had such impact on a specific individual’s eating practices at that specific time. The personal connotations explain why turning points varied in terms of impact and duration of the effect on eating practices. For example, from the nine turning points that Saskia mentioned, getting married improved her diet only for a short period of time, while the traumatic experience of being raped as a child still has a permanent impact on her eating behaviour. She identified this as the cause for developing emotional eating habits. In addition, the personal meaning (or situatedness) explains why similar experiences can have different effects on eating practices. For example, the reason why losing a job was such a significant turning point for Annie, but did not affect eating practices of some others, is that Annie perceived losing her job as the main cause for the termination of her relationship.

**Annie:** I was born in 1957 and until 1995 everything went smoothly. I never actually once thought that there was anything that could have an influence my eating. Well in 1995, I lost my job after 20 years and my relationship with my partner also fell apart. In response, I started to eat a lot [...] My partner and I also worked together. We used to see each other every day at work. And when that stopped, there were a lot of things that we didn’t talk about anymore. Most of it was connected with work and then at some point.. [...] I stopped seeing him every day, you know? Then it just becomes very different, yes.

Furthermore, turning points are highly contextual, and, therefore, should be interpreted in the light of the everyday life circumstances *at that time*. For example, Mark identified the health warning from his GP as a turning point. Yet, coincidentally, watching a TV show on reversing T2DM with lifestyle reinforced the effect of this turning point, and being retired facilitated the implementation of the dietary advice into practice.

Finally, the strong *temporal* influence of past experiences on present eating practices is needed for a meaningful interpretation as this serves as an overall ‘background’ for the turning points. Particularly, the undeniable influence of the childhood experiences on later food norms, cooking skills and eating practices. Both food-related and more general growing-up experiences influenced eating practices in later life. Typically, the participants grew up in large families under relatively financially deprived circumstances. A few had a happy childhood, but most had a disadvantaged childhood, in which they had to work hard and felt invisible to their parents. Some even experienced severe child neglect or abuse. Living in large families implicated that all food always had to be shared. Seemingly, being in charge of what, when, and how much to eat became important for making food choices in their later lives, and may explain why participants indulged themselves with snacks once they started to live on their own. Not being in charge of food choices and feeling unacknowledged may explain for some experienced resistance towards dieting: the ‘rules from the dietician’ limited (again) their freedom of choice.

**Freek**: A dietitian could tell me how much weight I would need to lose and this and that, but kind of authoritarian pressure doesn’t work for me.

**Dennis:** They [*health professionals*] don’t think along with you, but more in the line of: “Let me tell you what’s going to happen”, and that does not work for me; I’d just tell them to piss off.

Furthermore, most participants were accustomed to eating fresh and self-produced foods during their childhoods (processed foods were relatively scarce and expensive at that time in the Netherlands). They learned and were taught food-related skills by helping their parents with harvesting, preparing, and persevering foods. Eating fresh products and cooking similar dishes to what their parents cooked was considered ‘proper’ and healthy food.

**Freek:** [*My mother made*] all the old-fashioned traditional Dutch dishes; stamppot, kale with sausage, carrots, sauces, etc. We also slaughtered our own pigs and cattle ourselves, so there was plenty of everything. We had the potatoes ourselves, you had the fruit ourselves; all year round. [...] We only had to buy butter, a pack of sugar, and some flour. [...] In winter, apples were picked and peeled, and they were sent to the stone factory. There, they were dried and you could eat them on a winter’s day. [...] Everything was used. [...] And then you also knew what you ate. Do you remember what you eat now? It’s all stuff from abroad. People now don’t know anything about half the food they eat.

### Coping styles

Almost all participants experienced an ill-health confrontation turning point that led to reflectiveness on eating practices. However, how this affected actual eating practices in the present varied strongly among the participants: not all were able to eat in line with their new intentions. Broadly, two coping styles for healthy eating were distinguished: active and passive coping. In active coping, two subtypes are described: healthy coping and happy coping. The nuances within, and reasons for these coping styles are discussed in the next two paragraphs. Notably, of the participants with high SoC (> 35), most (*n* = 8/11) had an active coping style.

#### Active coping

Active coping is defined as cognitive and behavioural attempts to deal directly with stressors and their effects [69]. Two types of active coping were found that are referred to as ‘healthy’ coping and ‘happy’ coping. Healthy and happy coping have in common that the actual coping strategies are in line with deliberate decisions (intentions), however, the priority given to healthy eating in these decisions differed.

##### Healthy coping

Participants with a healthy coping style were able to realise their intentions to eat more healthily in everyday life. For some (Diane, Henk, Mark, Dennis, Tygo, Theo) this already led to weight loss and/or lower T2DM medication use, whereas others (Annie, Saskia and Jan) started with healthier eating only recently. All demonstrated an accurate assessment of the extent to which an unhealthy diet is indeed a threat by accepting their T2DM, and acknowledging their own influence on their health. They also internalised being a healthy eater as a part of their identity.

**Annie:** [*While describing the items of her food box; pictures of all sorts of healthy foods*] That is what I eat. And people who really know me will recognize me in this because I eat healthy nowadays.

Secondly, their coping strategies for challenges for healthy eating were more flexible and creative than the other participants. Firstly, they generally focused on what they *should* and *could* eat instead of what they *could not* eat. Their new eating strategies were characterised by eating *more* instead of less (e.g. either eating more frequently, more of protein-rich foods, more vegetables, or more high-quality foods) and by being creative with foods (e.g. replacing snacks with healthy alternatives; experimenting with new recipes).

**Dennis:** The thing with food is, as I have already mentioned, that if you have good food, you don’t need any sauce, so there won’t be any excess sugars.

In addition, they did not want to become obsessed about dieting and weight loss, and, therefore, lost weight rather slowly by changing diets gradually. Thirdly, they were actively involved in a trial-and-error process to understand their bodies better with the help of self-monitoring blood glucose.

**Dennis:** For now, my values are okay, my weight is going down, so I am satisfied. Then I will not continue to hurt myself with the thought of having to do this or that, or worry about what’s allowed or not. [...] If I do that, it becomes an obsession and I don’t want that.

**Saskia:** I want something that is entirely tailored to my needs – identify what makes me fat, like meat, so I can leave that out. I figured that out a bit and implemented it myself.

Lastly, almost all reached out for professional dietary help; either from a practice nurse/general practitioner or dietician. Notably, these participants reported pleasant and more positive experiences with their healthcare professional. Compared to the other participants, they felt trusted and supported by their healthcare professionals. They appreciated their healthcare professional’s clear communication and ability to connect to their personal lives.

**Tygo:** Well, I’ve been to a dietitian before [...], but they switched jobs a lot.. I have had three or four different ones there. And then the next one left and so on... That didn’t work. And then I started exercising here, and there was Fleur [*dietician*]. Then Adam [*fitness trainer*] said: “Do you want to go to a dietician?” I wanted to, so then I went two or three times and then she [*Fleur*] quit, then she went to Loenen [*another city*]! I said: [...] “I would like to continue working with you!”. And then we went to Loenen [...] That click that was there. If I have a good feeling with someone...

**Dennis:** Josien, [*practice nurse*] she talked about it and then she said she could also send me to a dietician. “Would you like that?” she asked. It wasn’t that I had to, but she really asked for my consent. “If you want, she can call you to make an appointment.” Well, I thought it wouldn’t hurt to try. And that whole conversation with the dietitian then also came back to Josien [...] You are not rushed along or anything. Because you get that a lot, those professionals who just become snappy if you don’t do as they ask.

However, there were individual differences in the level of satisfaction with, and attitudes towards current eating strategies. For Mark, Jan and Dennis, adhering to new eating strategies was easier than for Diane, Annie, Saskia, Henk and Tygo. The two extremes in this type of coping were Mark and Diane; while Mark seemed to be ‘effortlessly healthy’, Diane seemed more ‘miserably healthy’.

**Mark:** We eat a lot of vegetables, lots of fresh vegetables if possible. And in the evening I do eat eggs or nuts for 2 or 3 days a week instead of a sandwich. So, no bread on those days, that’s how I started this diet. And I feel very comfortable with that [...] She [*practice nurse*] always pushed for those medicines. But then those shows about diabetes were on television [*shows on national TV in which people improved their T2DM by making dietary changes*]. Then I thought: if it works that way with food, why wouldn’t it work me? I will just go for it and see what the results will be.

**Diane:** And then I stopped smoking last year and then I was at 109–110 [*kilos*]. And now I am a year further and I am around 105 [*whispering*]. So, I lost five kilograms in a year’s time .. even though my life [*style*] [...] this is very small, don’t you think? Five kilos in 1 year [...], I really don’t understand how that is possible. And if I were to have a bad week, I’d easily gain 3–4 k again.

Two clear differences between Mark and Diane were that Diane’s diet tactics were more restricted and less flexible, and that Diane changed her diet without the help of a professional due to prior negative experiences. Together, this could lead to a situation in which Diane’s frustration about healthy eating per se becomes a stressor.

##### Happy coping

Ria and Freek did not necessarily have the most optimal, healthy diets, and were also aware of this, but, nonetheless, were satisfied with their diets as they were and did not have a wish to improve their diets. It is important to emphasise that they were not careless about their diets: both were eating healthier compared to how they used to eat before. Yet, they were simply not willing to restrict themselves any further. Feeling good and being able to do the things that they wanted to were more important to them than improving dietary habits and losing weight. They were focused on the ‘here and now’, rather than worrying about their future health.

**Ria:** It is not important to me, eating. I mean, let me put it this way: I care very little at all for food.

**Freek:** Just do what you have to do and what makes you feel best. And that’s it in a nutshell [...] It’s the little things, but if you do it that way every day, you can do a lot.

Compared to the ‘healthy’ coping, the willingness of these participants for developing and considering new eating strategies seemed to be lower. They were more focused on preventing health complications by *remaining* on the same weight rather than actively losing weight. Their eating strategies were overall less drastic, more restricted (not buying snacks; limiting overall food intake) and less creative (e.g. using an air fryer and drinking diet drinks, but not developing new cooking skills) compared to ‘healthy’ coping.

**Ria:** No, I thought that I was [*eating healthily*], I’m satisfied with it [...] I will keep it up, if nothing else comes into my body. And that I get the answers of ‘yes you are doing good’ when I go to the GP check-ups.

#### Passive coping

Passive coping is defined as cognitive attempts to avoid actively confronting problems and/or behaviours to indirectly reduce emotional tension through such behaviours [69]. These participants (Mieke, Karin, Carla, Marja, Claudia and Robert) experienced worries about their health and the incongruency between their intentions and actual eating behaviours after experiencing a confrontational turning point caused by ill-health, yet, they struggled to realise their intentions to eat more healthily into everyday life. Multiple reasons attributed to this. Firstly, they had more difficulties in accepting T2DM and facing the reality of the disease. Self-monitoring blood glucose was, therefore, perceived as very stressful and was often avoided.

**Interviewer:** Has anything changed [*in how you view the disease*] compared to 10 years ago?

**Mieke:** No, no. I still think ‘oh, that one is sick’ [*points to empty seat on couch*]. Not me, you know.

Secondly, they expressed to a greater extent that healthy eating in combination with T2DM is complex, particularly adjusting medications to sudden dietary changes. In addition, they were more confused by conflicting information from healthcare professionals and family members and/or friends (e.g. are diet or low calorie products healthy or not?). They also did not know how to eat healthily without starving themselves and/or comprising their enjoyment of eating (i.e. lacking knowledge/cooking skills). Instead of thinking of new ways to handle difficult situations better (i.e. active coping), these participants eating strategies were characterised by being more restricted (e.g. sticking to a strict routine; eating the same things) and more avoiding (e.g. eating at home as much as possible).

**Marja:** In terms of food and drink, we just ate the same things as always, nothing special. You can cook nasi once, but then you bear in mind what you put into it. And that you would eat that once. We also never eat at a Chinese restaurant or anything.

**Mieke:** but it is often his job, [...], which always causes problems. So, now I’ve told him [*her husband*] if you have some event again, then I’ll stay home. [...] especially when it involves food, you know? If I have to hand over control to someone else. That I no longer have control of it myself. Yes, that, that’s difficult for me [...] Regularity is actually best for me. Just go to bed on time, eat on time, and get up on time.

Finally, they felt less supported by healthcare professionals and have disappointing experiences with dieting in the past (i.e. regaining weight after periods of dieting; unsupportive healthcare providers) which made them reluctant towards dieting.

**Claudia:** I lost a lot of weight years ago, at that time, I went to the dietitian and everything. [...] But when I stopped, it immediately came back again, so I said to myself that I wouldn’t do that anymore.

Instead, they try now to accept themselves as they are. This can be seen as a ‘last resort’: instead of changing diets, they changed their attitudes towards diet and health.

**Karin:** So ... yes. That’s why I just do it. I also have no idea if my sugar gets too high, what I have to do then.. how I can notice it or something. I don’t know that either [...] So.. I do actually find it a bit tricky. [...] And why you do it or not; I don’t think that my doctor would be concerned.

**Mieke:** I’m really getting sick of it. [...] And then I also said to Marijke [*practice nurse*] “I’ll just give up”. I’d say, take me as I am, even if that means I’m overweight. I don’t care anymore. [...] Do you understand? I mean, ... then you have peace with yourself. And maybe that’s when you will lose weight.

There was individual variation in the attitude towards this type of coping: some (e.g. Karin) were overwhelmed by worries, stress and emotions at the time of the study, however, most seemed to have been moved past this initial ‘crisis’ stage.

**Interviewer:** And what are things that make it easier for you to eat healthily?

**Karin:** Well.. I don’t really know that. I don’t find it easy at all. But it just has to happen. I have to be careful. If my sugar is too high then you will also have problems and I also think about being a mother where the father is no longer there for the children. I then think, yes, this mother should of course continue to live a little longer. Yes, it sounds a bit strange but that is of course how it is. That can make me a little bit emotional [*crying*].

## Discussion

Impactful, and unfortunately often bitter, life stories, rather than a lack of motivation or nutritional knowledge, explained the development unhealthy eating practices in people with T2DM and of low socioeconomic position. Adverse childhood experiences followed by an unequal share of hardship in later life deprived participants from developing strong psychosocial resources important for both managing emotional stress and healthy eating, paving the way to developing unhealthy eating practices. Indeed, a meta-analysis showed that adverse childhood experiences – especially neglect – increased the risk of T2DM by 32% [70]. The findings are also in line with a large body of evidence that demonstrates that suboptimal coping with stressful life events and negative emotions are associated with lower individual resilience [71], unhealthy eating [72, 73], weight regain [74–76], the onset of cardiovascular diseases [77] and T2DM [70], and suboptimal self-management behaviours [78–80].

The study identified moments when people are potentially more open for dietary change (confrontation with ill-health; becoming a parent, particularly when experienced child neglect; psychological therapy for treating depression), but also under which circumstances dietary change is more difficult or even impossible (losing a loved one, depression, suffering from pain, etc.). A confrontational health turning point was the most powerful turning point for healthy eating. The powerful impact for T2DM self-management of such an experience has also been observed in previous research [29, 30, 33, 34]. An important distinction between the present study and previous ones is that the present one adopted a life-course perspective, whereas the previous studies focused on a more defined period (i.e. T2DM diagnosis and onwards) [29, 30, 33, 34]. For example, one study identified experiencing coherence between newly adopted health behaviours and illness-related results as an important turning point for T2DM self-management [34]. Interestingly, in the present study, such illness-coherence experiences were not identified as turning points, but as consequences of the turning points for healthy eating. This does not mean that illness-coherence is irrelevant, in fact, insights of this nature were mentioned as useful for self-management by the ‘healthy’ copers. The present study complemented these insights by showing how someone’s relationship with food develops over time, and, importantly, that also non-disease and non-food related life experiences, as well as the availability or resources play a role in people’s attitude towards and coping strategies for self-management behaviours.

The period after a confrontational ill-health turning point is an opportune time for changing eating practices. However, it should be stressed explicitly that confrontational health turning points cannot be forced externally; they come from within the individual. A meta-analysis showed that confrontational health warnings are only effective if the receiver’s self-efficacy is already high, but, otherwise, have minimal and even negative effects for health behaviour [81]. Therefore, confrontational ill-health turning points are windows of opportunity for dietary change *if* health professionals recognise them at the right time and provide appropriate guidance for a self-reflective process. In addition, this study emphasised the importance of the timing of life-events: turning points for healthy eating only occurred when someone was not facing (too many) stressors at the same time.

Finally, the results show that people *can* turn back from turning points due to the fact that life – maybe particularly in socioeconomically disadvantaged conditions – is ever-changing in terms of (stressful) circumstances, physical (ill)health, and/or significant resources. For example, Robert regained weight due to Parkinson’s disease as it complicated his healthy lifestyle. Positively, an initial negative event for healthy eating such as childhood neglect can have a positive effect under different life circumstances (becoming a parent). In addition, participants were even at later age still motivated to change lifestyle behaviours, given the right conditions.

### Theoretical interpretations

**Fig. 2:**
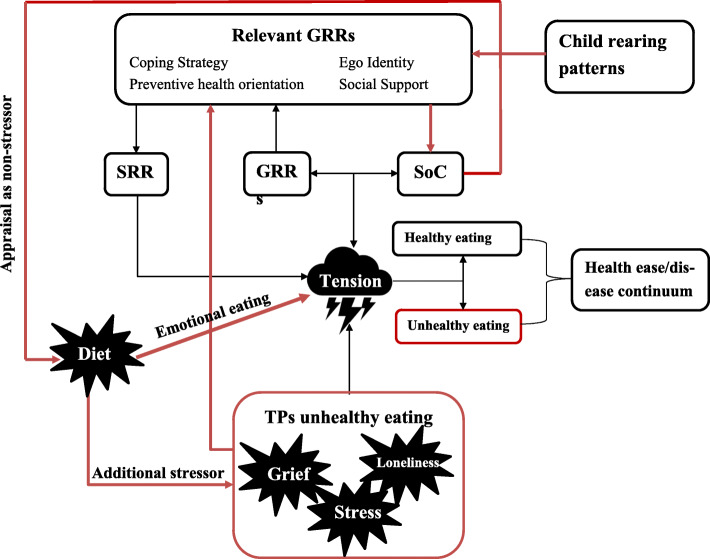
Proposed salutogenic explanation of the turning points (TPs) for unhealthy eating. Turning points for unhealthy eating caused an overload of stress(ors) that disturbed the emotional stability strongly. The SoC-GRR-SRR pathway’s full capacity was needed to handle the tension created, which necessitated appraising diet as non-stressor. In some, (unhealthy) eating was used for dealing with the tensions (i.e. emotional eating). Often this caused rapid weight gain which complicated the situation further because diet became then a stressors on top of the tension-overload. Child-rearing patterns are important for developing GRRs. Growing up in poverty, experiencing childhood neglect/abuse, not feeling acknowledged by parents for the unique human being they are, were early life conditions/experiences that hindered an adequate development of psychosocial GRRs. In addition, turning points for unhealthy eating affected psychosocial GRRs negatively (e.g. damaged ego identity; feeling unsupported), which weakened the SoC-GRR-SRR pathway, and complicated dealing with stressors further

Applying a salutogenic lens, turning points for **unhealthy eating** lead to overload of the SoC-GRR-SRR pathway whereas turning points for healthy eating were ‘SoC-strengthening’ experiences. The detrimental effect of turning points for unhealthy eating can be explained by the incapability to manage stress in combination with an unequal share of life adversities; healthy eating was no priority under such circumstances. There was so much tension that the pathway’s full capacity was required to handle the emotional aftermath of the turning points for unhealthy eating (Fig. 2**)**. This necessitated appraising diet as a comparatively small stressor or non-stressor. Even more troublesome, some individuals used eating to cope with the tension resulting from the turning point, which can lead eventually to a situation in which diet becomes an additional stressor on top of the already unmanageable tension. Turning points for unhealthy eating seemed also to affect psychosocial GRRs negatively (e.g. damaged ego identity due to losing a job; feeling unsupported during times of grief). This complicated dealing with stressors further.

**Fig. 3:**
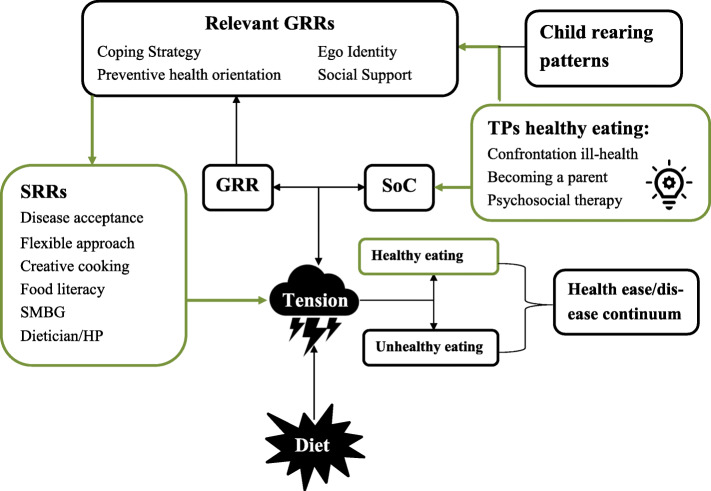
Proposed salutogenic explanation of turning points for healthy eating. Turning points for healthy eating only happened when someone was not facing other significant stressor(s) at that time. The effect of turning points for healthy eating can be interpreted as ‘SoC-strengthening’ as these experiences changed outlooks on life and induced reflexivity on how current eating practices may comprise future goals. By this, diet became more of a priority (*meaningfulness*), which led to insights on what needs to be changed (*understandability*) and what was needed to realise this (*manageability*). Turning points for healthy eating also affect psychosocial GGR positively, which strengthened the overall SoC-GGR-SRR pathway. A psychosocial GRR that seemed particular relevant for realising diet changes is coping strategy. If this GRR is well-developed, it facilitates developing coping strategies for specific situations/challenges, which requires in this case, understanding the importance of healthy eating and personal challenges within in this, making plans to overcome the challenges, anticipating challenging situations, and being flexible with this. Logically, this facilitates identification and use of SRRs relevant for realising dietary intentions

Turning points for **healthy eating** strengthened the SoC-GRR-SRR pathway as they involved reflexivity and self-redefinition, an observation in line with previous research [25, 28–30, 82, 83]. It changed participants’ outlooks on life and induced reflexivity on how current eating practices may comprise future goals. By this, diet became more of a priority to participants (i.e. healthy eating as a resource for health/life; *meaningfulness*) and it gave them insights into what needed to be changed (i.e. old habits, emotional eating behaviours; *understandability*) and what is needed to realise this (i.e. seeking help form a professional; *manageability*). Turning points for **healthy eating** also seemed to affect psychosocial GGR positively and directly (ego identity, social support), which strengthened the overall SoC-GGR-SRR pathway (Fig. 3). Previous research also suggested that self-examination (introspection and reflection) is fundamental for enhancing SoC [84] and adopting active coping [85]. Remarkably, most participants with high SoC score had indeed an active coping style.

However, even though almost all participants experienced a turning point for healthy eating, only a part of the participants seemed to be acting in line with dietary intentions (i.e. active coping) and a smaller group seemed to adhere to dietary guidelines (i.e. ‘healthy’ copers). This argues that self-examination alone may be a start for health behaviour change, but actual change requires also the presence of (other) well-developed internal and external GRRs/SRRs. Difference between passive- and active coping can be largely explained by difference in the extent that GRRs and SRRs were developed. Important GRRs were coping strategy, preventive health orientation, ego identity and social support. Important SRRs were disease acceptance, a flexible approach to eating, creative cooking, food literacy, self-monitoring blood glucose, and a supportive dietician/healthcare professional (with the ability to connect on a personal level). In active coping, these GRRs and SRRs were used to deal with the tensions created by the turning points. This enhanced their capability to eat in line with their intentions and develop effective and flexible diet strategies.

Participants with passive coping on the other hand had difficulties in successfully overcoming the initial emotional consequences of turning points and incorporating active coping strategies. Passive coping seemed not so much the result of deliberate decisions, but of frustration caused by the incapability to implement nutritional advice successfully into new eating strategies. Among participants with passive coping, feelings of anxiety, confusion and being a failure were more profound compared to participants with active coping. SoC and psychosocial GRRs might be developed to a lesser extent, which may explain the less frequent (use of) SRRs for healthier eating (manageability) and the perceived complexity of healthy nutrition (comprehensibility). They also felt less supported by healthcare professionals, so in a way, participants with passive coping were lacking this important and highly valued SRR of participants with active coping. Lacking well-developed GRRs and SRRs necessitated suppressing fears/worries and avoiding confrontation with T2DM in order to cope with the tensions caused by confrontational turning points.

Happy coping can be easily misinterpreted as having less developed SoC and/or GRRs as well, however, this type of coping seems not so much the results of incapability to implement nutritional advice, but rather of not prioritising (physical) health to the same level as in ‘healthy’ coping. Other aspects of life (work/hobbies, family) were simply more meaningful to these participants. Prioritising diet more than they already do, would possibly interfere with their quality of life.

A particularly important SRR for the individuals with ‘healthy’ coping was a flexible approach to eating. Adopting a positive and flexible attitude also has been identified previously as an important resource for individual resilience for coping with T2DM self-management [71], diet [62], and academic barriers [86]. In addition, a flexible approach has been associated with successful weight loss and maintenance [42, 73, 76, 87–92]. The participants with passive coping showed an approach to eating was rigid and restrictive rather than flexible. Adopting a flexible approach to eating is challenging, especially for individuals with T2DM who have an eating history that includes repeated unsuccessful weight loss attempts, emotional eating and eating beyond physical satisfaction [93].

### Strengths and limitations

An important strength is the use of timelines and food-boxes, because these are easy, informal and accessible tools that facilitate a quick establishment of a trust relationship between the participant and the researcher. Participants easily opened up and enjoyed being part of the research. They felt someone was listening sincerely, and the interview gave them new personal insights. One participant even explained in the final phone call that the interview in itself was a turning point in a way. In our experiences, the current methodology led to richer data compared to structured interview methods that addressed eating practices and health behaviours more directly. Another related strength of the methodology is that participants *themselves* identified what they considered to be a turning point by preparing the timeline in advance, therefore, the study was closely connected to the participant’s lived experiences. In previous research, the definition of which experiences were turning points and which were not seemed more part of the analysis, and thus, more dependent on the researchers’ interpretation [25, 82, 83].

Yet, an important practical limitation of the present methodology is that it is more time-consuming regarding both execution and analysis. Secondly, this study did not include measurements on actual food intake and HbA1c (glycated haemoglobin) and blood glucose levels were self-reported, therefore, no firm statements could be made as to what extent the participants were truly eating in line with dietary recommendations. Some of the ‘healthy’ copers managed their glycaemic measurements in line with recommended targets, others did not. However, this should not be interpreted necessary as social desirability, because multiple factors (including genetics, age) influence glycaemic control [94, 95]. Instead, how people talked about their current eating practices was key for the categorisation of coping styles.

Regarding externally validity, it can be argued that the participants were of medium-low rather than of low-socioeconomic position. SoC values were also relatively high. In addition, the present study focused on native-Dutch. The onset of T2DM is generally at an earlier age in migrant-Dutch [96, 97], and socioeconomic position plays a markedly different role in explaining diet quality among migrant-Dutch people [98]. Hence, future research on turning points for eating practices in cultural minorities is needed. Furthermore, the focus of this study was on eating practices, therefore, no statements can be made as to whether these findings apply to other T2DM self-management behaviours or not. Future research that is focused on multiple aspects of T2DM self-management is desirable. Finally, it was difficult to recruit sufficient participants. The participants in this study may have had different viewpoints to those who could not be reached by the recruiters or refused to participate. Nevertheless, this exposes another important point requiring scientific attention: effective ways to reach and engage individuals of (the) low(est) socioeconomic position in research [99].

## Conclusion

This study demonstrates the consequences of the social environment for healthy eating over the life-course. The findings imply that individual differences in coping strategies for healthy eating are not the result of specific experiences or personal factors, but of a reflective, positive attitude towards life, and the presence of psychosocial (general and specific) resources. Healthy eating has the potential to improve long-term health, but the exploitation of that potential requires self-examination and supportive psychosocial resources. A stress-free state-of-mind, a flexible approach to eating, and feeling supported seem crucial in this. Overall, the findings are in line with previous research, stating that healthy eating is associated with an internal motivation, autonomy, self-efficacy, flexible dietary strategies, social support, effective stress management, and overall more psychological strength and stability [73, 100–104].

Therefore, especially individuals with T2DM with more passive coping might benefit from a healthcare system that is dedicated to empowerment of individuals by involving them actively in learning trajectories focused on reflexivity, self-examination, psychosocial well-being and social support. Crucial for this is that healthcare professionals are equipped with the right skills and sufficient time to do so [102, 105]. While acknowledging the complexity of what such a learning trajectory should entail exactly and the many institutional- and practical obstacles in this, incorporating reflective tools, such as timelines and food-boxes, may be a relatively easy first step towards creating a more empowering healthcare system. In addition, dietary therapy should aim at making healthy eating and cooking uncomplicated and enjoyable. Indeed, a growing body of research suggest that *positive* emotions – independent of negative emotions/stress – are associated with lower cardiovascular morbidity and mortality via suggested indirect (i.e. improved health behaviours) and direct physiological mechanisms (i.e. including neuroendocrine-, inflammatory-, immunological- and cardiovascular systems) [106]. Finally, the research shows that targeting at the right moment in life may be important for the success of a dietary therapy. More research that adopts a life-course perspective to illuminate the interaction between turning points and eating practices (and other self-management behaviours) is recommended.

## Data Availability

The datasets generated and/or analysed during the current study are not publicly available in accordance with protection of confidentiality and privacy, but are available from the corresponding author on reasonable request.

## Abbreviations

T2DM: Type 2 diabetes mellitus
SoC: Sense of Coherence
GRR: General Resistance Resource
SRR: Specific Resistance Resource
IPA: Interpretative Phenomenological Analysis

## Footnotes

1 These participants indicated to be willing to participate, but after the initial phone call, it became clear that their life situations at that time would make it severely complicated to schedule the interviews. Hence, after mutual agreement, these two individuals were put on a reserve list instead. Once data saturation was reached, they were informed that participation was no longer necessary.
